# Stress levels, Alexithymia, Type A and Type C personality patterns in undergraduate students

**Published:** 2010-05-25

**Authors:** A Lală, G Bobîrnac, R Tipa

**Affiliations:** ‘Carol Davila’ University of Medicine and PharmacyRomania

**Keywords:** stress, alexithymia, type A personality, type C personality, undergraduate students

## Abstract

**Introduction:** Since there have been a number of empirical observations that may lead to the conclusion of an increasing rate of risk behaviors in Romanian students, such as aggression, over–competitive conduct and lack of collaboration, immorality, peer pressure and even an increasing rate of suicide, and suicide attempts, we have undergone a study to indentify if there is a high rate of risk type personality patterns that may lead to these deportments.

**Material and methods:** We have selected a total number of 500 students from the three largest universities in Bucharest, Romania–‘Carol Davila’ University of Medicine and Pharmacy (UMF), Bucharest Polytechnics University (UPB), and the Bucharest Academy of Economical Studies (ASE). All subjects received a questionnaire containing four diagnostic tools and several demographics questions. We have chosen the Twenty Item Toronto Alexithymia Scale (TAS20), the Jenkins Activity Survey (JAS–13) and the Anger–In Questionnaire for type C personality pattern.  We have also added the Columbia stress analysis questionnaire for the evaluation of stress levels and coping capacity at the moment the subjects were interviewed.

**Results:** Columbia stress survey results confirm that there is a high stress level among students of all universities, but a more detailed stratification by university, gender and analyzed factor shows a very high F factor and T factor positive responses. Alexithymia, Type A and Type C personality patterns show a much higher prevalence than the general population, especially in medical students. We have found higher frequencies in men for all of the three studied parameters.

**Conclusions:** Approaching alexithymia and type A behavior both by cognitive methods and by assessing and addressing consequential risk factors should become an issue among universities.

## Introduction

Since there have been a number of empirical observations that may lead to the conclusion of an increasing rate of risk behaviors in Romanian students, such as aggression, over–competitive conduct and lack of collaboration, immorality, peer pressure and even an increasing rate of suicide and suicide attempts, we have undergone a study to indentify if there is a high rate of risk type personality patterns that may lead to these deportments.

Alexithymia, first described by Peter Sifneos in 1973, is a personality trait placing individuals at risk for different physical and psychological disorders. It is not classified as a mental disorder by the DSM Ⅳ manual, but it has a well known correlation with low patient compliance and certain personality disorders [1], substance abuse disorders [2], sexual disorders [3], physical diseases such as hypertension [4], fibromyalgia[5], and psychosomatic disorders such as headaches, back pain, inflammatory bowel disease and functional dyspepsia. Also, modified immune responses have been found–low NK T–cell activity because of accentuated inhibition by a hyperactive hypothalamic–pineal–adrenal system which has been linked to alexithymia.[10]

Alexithymia is defined by 4 main characteristics [5]:

1. Difficulty in identifying feelings and distinguishing between feelings and the bodily sensations of emotional arousal;
2. Difficulty in describing feelings to other people;
3. Constricted image processes, as evidenced by a paucity of fantasies;
4. A stimulus–bound, externally oriented cognitive style.

Several theories and studies suggest that alexithymia has a genetic basis, but environmental causes have also been described; it has been suggested that persons suffering from traumatic brain injury are six times more likely to develop the disorder during their lifetime.[6]

Type A personality is a pattern conglomerating persons with a high competitive spirit, irritability, aggressiveness, hostility, vengeful and with a strong feeling of urgency, with a strong ambition and an impulsive conduct [7]. Having a strong need for praise and lacking satisfaction for their work, individuals who exhibit type A personality pattern are veritable ‘stress collectors’, prone to developing psychosomatic disorders.[8]

There are several correlations between type A personality pattern and different physical and psychological disorders. In the medical field, this pattern has been correlated with high plasma triglycerides and cholesterol levels, hyperinsulinemia, reduced coagulation times, high plasma cortisol levels, and low growth hormone levels.[9] Psychological disorders such as anxiety and depression, or negative conducts like frustration, hostility, lack of adaptation and low coping with stress [10] are found to be consequential to  this type of personality. In addition, there is a strong theoretical link between this pattern and coronary heart disease, but newer studies and hypotheses tend to affirm the contrary [11].

Type C or ‘anger in’ personality, considered the opposite of type A, was first described by Temoshok, as a repressive and vigilant personality pattern [12]. It has been marked as a risk factor form of cancer, and there is a special correlation between type C personality and the occurrence of breast cancer. 

The main characteristics are:

1. Strong defense mechanisms leading to incapacity of verbalization and recognition of the subject's own negative emotions;
2. Secondary negative reactions such as feelings of hopelessness and uselessness;
3. Lack of self–control in stress situations.[8]

There is an association between type C personality and alexithymia.

## Material and methods

We have selected a total number of 500 students from the three largest universities in Bucharest, Romania – ‘Carol Davila’ University of Medicine and Pharmacy (UMF), Bucharest Polytechnics University (UPB) and the Bucharest Academy of Economical Studies (ASE). The selection of groups was made both by the number of students in the university and by the study field of that university, while trying to select groups that have very different study curricula and environments. 

All subjects received a questionnaire containing four diagnostic tools and several demographics questions. We have chosen the Twenty Item Toronto Alexithymia Scale (TAS20), the Jenkins Activity Survey (JAS–13) and the Anger–In Questionnaire for type C personality pattern.  We have also added the Columbia stress analysis questionnaire for the evaluation of stress levels and coping capacity at the moment the subjects were interviewed. The questionnaire was to be completed at home and returned the next day, in order to avoid the social influence factor and time pressure.

The TAS–20 scale is a revises version of the Toronto Alexythimia Scale, and is now the most widely used instrument to measure alexithymia levels [13]. The TAS–20 has 3 factors including: difficulty in identifying feelings and distinguishing them from bodily sensations, difficulty in  describing feelings to others, and externally oriented thinking. Preliminary evidence of reliability and factorial validity has been established [14].

The Jenkins Activity Survey is one of the most widely used methods of assessing Type A behavior. The Jenkins Activity Survey is a psychometric survey of behavior and attitude designed to identify persons showing signs of Type A behavior [15]. Reliability estimates for the JAS Type A scale appear to be adequate. Estimates of item reliabilities derived from squared multiple correlation coefficients ranging from 0.27 to 0.75; with the reported coefficient of 0.85 for the Type A scale. Test–retest reliability estimates generally range between 0.60 and 0.70 for retest intervals starting from six months to four years. However, most of the correlations are based on significant modifications in the items of later versions of the JAS.[16]

The Anger–In questionnaire is an 8 question multiple choice auto–administered survey used for assessing Type C suppressor, stoic, and ‘denier of feelings’ behavior. [17]

The Columbia stress surveys consist of three 10–item subscales measuring stress vulnerability due to frustration (F), vulnerability to over–soliciting (S) and stress due to pressure of time or constraint (T) and a 14–item recovery scale that measures the efficacy of stress–adapting and coping techniques

We have also added three original questions in order to establish some issues of the responder's personal and subjective perspectives on stress, expectancies and overall quality of life.

**Demographics:**  we have used several demographics in order to stratify groups. These were university (UMF, UPB or ASE), gender, study year and age. Moreover, we have asked the respondents to fill out their e–mails on a post–it if they want to receive results via e–mail, with a stipulated mark in the operational protocol that the post–it will be removed from the questionnaire right upon grading, in order to keep the anonymity of the respondents. We kept in mind the fact that none of the respondents was an acquaintance of the interviewers or the study authors.

**Analysis:** the analysis was made by using Epi Info ver. 3.5.1 software obtained from the Center for Disease Control (CDC) website. Only statistics and linear regression methods were used.

**Responder groups:** From the 500 issued surveys, 14 were declared invalid due to the missing information or demographics. The gender prevalence of responders stratified by university is almost the same as the general percentage shown for each university, although official data could not be obtained from the schools. Female gender among responders is higher in UMF (65.7%) and ASE (74.1%) and male gender is considerably higher in UPB (88.7%) (Table 1). General gender percentages among responders are 56.8% female and 43.2% male. 

Responder age stratified by university (Table 2) shows an average age of 19–20 years old, corresponding with the 2nd and 3rd year of study. Keeping in mind that Romanian undergraduate studies last for 6 years in medicine (UMF), 4 to 5 years in engineering at the Polytechnics University (UPB) and 3 years in economical studies (ASE), we can conclude that the results are valid for the middle to high years of study. We have selected this group in order to be able to correlate the results with the undergraduate study activity, although as results will show, we have not found a strong relation between the year of study and the percentage of positive test results for the three surveyed parameters. 

**Table 1:** Gender prevalence among subjects stratified by University. CL=95%

University	F	M	TOTAL
ASE count	126	44	170
ASE percentage	74,1%	25,9%	100,0%
UMF count	138	72	210
UMF percentage	65,7%	34,3%	100,0%
UPB count	12	94	106
UPB percentage	11,3%	88,7%	100,0%
TOTAL count	276	210	486
TOTAL percentage	56,8%	43,2%	100,0%

**Table 2:** Responder age and frequency stratified by university. CL=95%

Age	ASE	UMF	UPB
18	5,90%	4,80%	1,90%
19	68,20%	32,40%	60,40%
20	16,50%	21,00%	24,50%
21	3,50%	15,20%	0,00%
22	3,50%	19,00%	3,80%
23	2,40%	7,60%	9,40%
Total	100,00%	100,00%	100,00%

## Results

**Columbia stress survey results** (Table 3) confirm that there is a high stress level among students of all universities, but a more detailed stratification by university, gender and analyzed factor (Table 3) shows a very high F factor and T factor positive responses. There is no statistical correlation between gender and positive results (r^2^=0,03 for a p value of 0.029 and 95% CL), but there is a correlation between the low percentage of a gender among university students and positive results for high stress levels. 

**Table 3:** Columbia F, S and T factors positive result percentage, stratified by university and gender, CL=95%

	Female responders			Male responders		
	F factor	S factor	T factor	F factor	S factor	T factor
ASE	85.70%	12.70%	63.50%	90.9%	18.2%	45.5%
UPB	83.3%	50.00%	83.3%	71.7%	36.2%	68.1%
UMF	91.3%	26.1%	75.4%	83.3%	36.1%	55.6%

There is a noticeably high percentage of F (frustration) and T (time pressure) factors grading the main characteristic of a student's life, since all three universities have a consuming curriculum that forces the student's memory (medicine) or his logic– mathematic intelligence (engineering and economics) to the fullest in a very short amount of time. The T factor is strongly correlated with type A personality pattern [18]. Relatively low vulnerability to over–soliciting shown by S factor positive results may be correlated with the middle–end undergraduate study period of the selected group and the adjustment to the stressing conditions. Recovery scores for the selected group are relatively low, with a higher sum of F, S and T factors score than the recovery score in 43.6% of the subjects.

**Alexithymia, Type A and Type C personality patterns** show a much higher prevalence than the general population, especially in medical students. We have found higher frequencies in men for all of the three studied parameters (Table 4, Table 5 and Table 6). 

**Table 4:** Frequency of Type A, Type C and Alexithimia positive parameters in responders, 95%CL

Personality pattern	Freq. in men	Freq. in women	General freq.
Type A	28.2%	24.6%	25.9%
Type C	26.9%	9.4%	16.8%
Alexithymia	32%	21%	26.1%

**Table 5:** Variability of anger in, type A and alexithymia results stratified by university. 95% CL

	Type C positive	Type A positive	Alexithymia positive
UMF	21%	30.8%	24.5%
UPB	20.4%	2.6%	36.0%
ASE	9.4%	18.6%	21.2%

**Table 6:** Variability of type C, type A and alexithymia positive results stratified by university and gender. 95%CL

	UMF M	UMF F	UPB M	UPB F	ASE M	ASE F
Alexithymia	30.6%	20.3%	34.0%	66.7%	31.8%	17.5%
Type A	38.2%	27.5%	31.9%	16.7%	4.5%	22.2%
Anger In	37.1%	13.0%	19.1%	33.3%	27.3%	3.2%

Alexithymia general population positive values range between 11.8% among men and 8.1 percent among females [19], but they may rise according to age to a level of 31% in third age men and 29% in third age women. Gender differentiation is of no statistical value in young subjects, but percentage differences become noticeable when comparing old age subjects[19].  Our results set a general prevalence of 26.1% (95% CL), higher than the average for the studied age interval [19].  

Type A personality pattern levels are also higher than the general population and so are type C personality types. Although there are studies positively correlating the female gender with stress symptoms and patterns [20], our study shows a higher prevalence in men for all studied risk factors (Table 4). The general prevalence for this parameter was of 25.9%, with 24.6% in women and 28.2% in men (95% CL).

Type C personality pattern was found to have a general frequency of 16.8%, but, since there is no available study to set a general population limit, we cannot set a standard for comparison. The values averaged between 9.4% in women and 26.9% in men responders; still keeping a higher value for the male group.

## Conclusions

Stress levels, alexithymia and type A personality are higher in medical, engineering and economy students than in the general population. We have found different averages of type A, type C and Alexithymia results stratified by gender, male subjects being more affected than women. Commenting on university stratification, we can conclude that medical students at the middle and end of their 6–year study program have higher percentages for stress parameters and risk behaviors (type A, type C and alexithymia) than engineering and economy students who have a 4, respectively 3–year study program. We have to take into consideration that these personality types are patterns that have been positively correlated with high–risk conduct, especially when identified at such a young age and without the appropriate measurements taken.

These behavior patterns have a great importance since they have been correlated with several functional psychological parameters. Stress symptoms have been positively correlated with ineffective coping and Type A behavior, stress symptoms of Type A subjects were significantly predicted by the insufficient use of effective coping styles and deficiencies in the general mood component of emotional intelligence [20]. Type A behavior has also been positively correlated with the chronic fatigue syndrome [21], cardiovascular disease [22] and has been determined to be a risk factor for anxiety and depression in medical students and medical residents [23]. 

This behavior is especially important since, lately, studies have emphasized the role of the ‘hostility factor’ both in psychological and physical health issues. Studies have shown that subjects, who had a high score for the hostility factor, in the personality assessment inventory, during student life, were five times more susceptible of death before the age of 50 than the control group [24]. A recent classification suggests there is a ‘toxic nucleus’ of Type A behavior composed of four factors: A (ambition), S (restlessness), H (hostility) and J (workaholic attitude); of these, the H factor has been proven to be the cardiovascular disease risk factor.[25]

Alexithymia as a risk factor has been correlated with alcohol related risk perception among undergraduate students [26] and with an increase in smoking behavior [27], although these conclusions are still controversial. Other studies conclude that alexithymia was closely linked to concurrent depressive symptoms, and that depressive symptoms may act as a mediator between alexithymia and psychiatric morbidity.[28] 

Hostility and alexithymia are also correlated, concluding that alexithymia moderated, and partially mediated, the relationship between bullying and deliberate self–harm (DSH). Bullying and DSH significantly co–varied when alexithymia was moderate or high, but not when the participants' alexithymia was low, as shown in a study among adolescents.[29]

As an overall conclusion of research until currently, alexithymia was significantly associated with lower health–related quality of life, even independently of other variables[30]. Approaching alexithymia and type A behavior both by cognitive methods and by assessing and addressing consequential risk factors should become an issue among universities.
